# Robust policy evaluation from large-scale observational studies

**DOI:** 10.1371/journal.pone.0348228

**Published:** 2026-06-30

**Authors:** Md Saiful Islam, Md Sarowar Morshed, Gary J. Young, Md. Noor-E-Alam

**Affiliations:** 1 Mechanical and Industrial Engineering, Northeastern University, Boston, Massachusetts, United States of America; 2 Center for Health Policy and Healthcare Research, Northeastern University, Boston, Massachusetts, United States of America; 3 D’Amore-McKim School of Business, Northeastern University, Boston, Massachusetts, United States of America; 4 Bouvè College of Health Sciences, Northeastern University, Boston, Massachusetts, United States of America; Public Library of Science, UNITED KINGDOM OF GREAT BRITAIN AND NORTHERN IRELAND

## Abstract

Under the current policy decision making paradigm we make or evaluate a policy decision by intervening different socio-economic parameters and analyzing the impact of those interventions. This process involves identifying the causal relation between interventions and outcomes. Matching method is one of the popular techniques to identify such causal relations. However, in one-to-one matching, when a treatment or control unit has multiple pair assignment options with similar match quality, different matching algorithms often assign different pairs. Since all the matching algorithms assign pairs without considering the outcomes, it is possible that with the same data and same hypothesis, different experimenters can reach different conclusions creating an uncertainty in policy decision making. This problem becomes more prominent in the case of large-scale observational studies as there are more pair assignment options. Recently, a robust approach has been proposed to tackle the uncertainty that uses an integer programming model to explore all possible assignments. Though the proposed integer programming model is very efficient in making robust causal inference, it is not scalable to big data observational studies. With the current approach, an observational study with 50,000 samples will generate hundreds of thousands binary variables. Solving such integer programming problem is computationally expensive and becomes even worse with the increase of sample size. In this work, we consider causal inference testing with binary outcomes and propose computationally efficient algorithms that are adaptable for large-scale observational studies. By leveraging the structure of the optimization model, we propose a robustness condition that further reduces the computational burden. We validate the efficiency of the proposed algorithms by testing the causal relation between the Medicare Hospital Readmission Reduction Program (HRRP) and non-index readmissions (i.e., readmission to a hospital that is different from the hospital that discharged the patient) from the State of California Patient Discharge Database from 2010 to 2014. Our result shows that HRRP does not have a causal relation with the increase in non-index readmissions. The proposed algorithms proved to be highly scalable in testing causal relations from large-scale observational studies.

## Introduction

This is a revised submission of [1]. After publication of this article [1], we notified PLOS One of an error in the p-value calculation, affecting Fig 4 and related text in the Experiment and result section of [1]. Following review by a member of the Editorial Board, the PLOS One Editors concluded that the conclusion drawn from applying the mathematical model to the large-scale validation dataset is incorrect. However, the evaluation of the model’s scalability remains sound, and the study’s methodological contribution is unaffected. To address the above errors, PLOS One has decided to retract [1], and the PLOS Publication Ethics team has invited us to submit a revised version of [1] for republication. In this revised version, we have corrected the p-value calculation, updated Fig 4, and revised the necessary texts in the Abstract, Experiment and result section, and Conclusion.

Effective and evidence-based public policy decisions aim to manipulate one or many socio-economic variables and analyze their impact on the desired outcomes [2]. The impact assessment is not associational but causal [2,3] which requires an understanding of the counterfactual—the difference in outcomes with or without the presence of the policy [4]. This is also true for any post policy evaluation [2]. A policy maker may design multiple policies and calculate the causal quantities including the effect of the proposed policies on different recipient groups, effects over time, possible trade-offs between competing goals, and, finally, choose the optimal policy [5]. The gold standard approach for calculating those causal quantities is conducting a randomized experiment [6–9]. In a randomized experiment, the experimenter will assign observations to either treatment or control group randomly; this randomness can avoid bias and eliminate confounding effects of covariates and thus can achieve unbiased estimation of treatment effects. In this case, a possible association between treatment and outcome will imply causation. However, many studies in health care, social science, economics, and epidemiology cannot be designed as a randomized experiment due to legal or ethical reasons. Randomization can also be impractical, time consuming, or very expensive. Hence, in most such cases experiments are performed on data that are collected as a natural process. Such experiments are called observational studies (also referred to as natural experiments or quasi-experiments) [10] and can be implemented in a prospective (collecting sample data as natural observation over time) or retrospective (experimenting on already collected data) way.

Making causal inferences from an observational study lacks the experimental elements of randomization on all possible background covariates (the observed and unobserved characteristics of a sample unit) [11,12] and are prone to bias and systematic confounding on covariates. However, with proper understanding of the underlying process and careful control of non-randomized data, it is possible to make a reasonable estimation of the causal effect [6]. Researchers have been utilizing matching methods for identifying causality since the 1940s [11] and it is one of the most popular methods. It was used or noted in as many as 486,000 academic articles involving causal inference (see S1 File). Matching methods examine the possibility of restoring or replicating properties of randomization based on the observed covariates [11]. In fact, matching attempts to retrieve the latent randomization within the observational data [13]. Being true to its name, matching methods aim to find a control group that is identical to the treatment group in terms of joint distribution of the observed covariates. As discussed by Stuart [11], and Zubizarreta [14], matching the empirical distribution of the covariates has several significant advantages. For example, matching forces the experimenter to closely examine the data, check the common support on the covariates, and assess the quality of inference. Even though the matching process can be complex, the outcome analysis is often done with simple methods [15]. For instance, the Rubin Causal Model (also known as Potential Outcome Framework) estimates the causal effect as the difference of expected outcomes between the control group and the treatment group [16]. Due to its simple architecture and other attractive properties (see [11,14,17]), matching has been used to make policy decisions in health care [18–21], education [22,23], economics [24], law [25], and politics [26].

In this paper, we adopt a robust methodology recently proposed by Morucci *et al.* [27] and extend it to accommodate causal inference from big data observational studies. We show the efficiency of the proposed methods by evaluating the impact of the implementation of the Medicare Hospital Readmission Reduction Program (HRRP) [28] on non-index readmissions—readmission to a hospital that is different from the hospital that discharged the patient.

## Motivation and contribution

### Motivation

**Fig 1:**
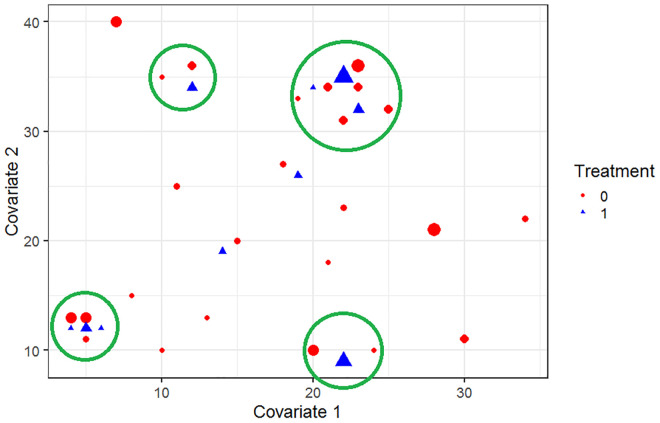
Uncertainty due to multiple pair assignment options. Shapes and Colors represent the treatment status and variations in size represent the difference in outcomes.

The objective of the current one-to-one matching paradigm under the potential outcome framework is to find pairs (*t*,*c*) between samples *t* from treatment group T and *c* from control group C. A pair (*t*,*c*) is assigned in such a way that *t* and *c* are the same or very similar on a specific, pre-determined set of covariates **X**: {(t,c):t≃c|𝐗;t∈T and c∈C}. Over *t*he years, researchers developed a wide array of algorithms to find such pairs, for example, Propensi*t*y Score matching [15], Mahalanobis Distance matching [15], Nearest Neighbour Greedy matching [29], Coarsed Exact Matching [30], and Genetic matching [31] are among the most popular algorithms. All these algorithms (including those not listed here) disregard the outcomes $\left(Y_{t}^{1},Y_{c}^{0}\right)$ of corresponding pairs (*t*,*c*) in the assignment process. Though the matching process reduces bias in treatment effect estimation, disregarding the outcomes in the assignment process introduces a new source of uncertainty. If a sample t∈T has multiple possible pair assignments $\left\{c_{1},c_{2},\cdots ,c_{n}\right\}\in C$ and have similar covaria*t*e balance but different outcomes (i.e., $Y_{t}^{1}-Y_{c_{1}}^{0}\neq Y_{t}^{1}-Y_{c_{2}}^{0}\neq \cdots \neq Y_{t}^{1}-Y_{c_{n}}^{0}$), by assigning pairs without considering the outcomes, an experimenter can estimate multiple degrees of causal effect (one for each possible assignment). Similarly, a sample from control group c∈C can have multiple possible assignment options $\left\{t_{1},t_{2},\cdots ,t_{n}\right\}\in T$. A possible scenario is presented in Fig 1 where within each circle we have multiple pair assignment options with almost similar match quality but different outcomes (outcomes are presented as the size of the data points). In such cases, different experimenters using different matching algorithms can get different pairs, hence, their causal effect estimates and conclusions on the experiment can be different. It is possible that two researchers having the exact same hypothesis and using the exact same data but with different matching algorithms reach completely opposite results due to this uncertainty. This problem is exacerbated for studies involving big data as we may have more pair assignment options. Therefore, making policy decisions in health care or any other field by using the matching method that disregards uncertainty due to pair assignments can lead to erroneous conclusions.

In 2012, Congress adopted HRRP as part of the Patient Protection and Affordable Care Act (PPACA) [28] to increase quality of care and reduce hospital readmission rates. HRRP penalizes hospitals when patients with certain clinical conditions (i.e., pneumonia, acute mayocardial infraction (AMI), congestive heart failure (CHF)) who have been discharged are readmitted within 30 days. The index hospital is always penalized even if the patient is readmitted to a different hospital (non-index hospital) [28]. Though readmissions to the index hospital are following a decreasing trend over the post HRRP periods, non-index readmissions are increasing [32,33]. This increase in the non-index readmission rate—approximately one fifth of all readmissions for Medicare patients [32,34]—creates suspicion that hospitals are possibly discouraging patients from readmission to avoid penalties introduced by HRRP. Moreover, a recent study identified that non-index readmissions are associated with higher odds of in-hospital mortality and longer length of stay [35]. Therefore, we aim to identify whether HRRP has a causal relation to the increase in non-index readmission. Finding such causal relation involves analyzing a large volume of health care data and matching method would be vulnerable to the uncertainty discussed above. The robust method proposed in [27] to handle such uncertainty requires solving multiple Integer Programming (IP) models (a minimization and a maximization problem) iteratively. Using state-of-the-art integer programming solvers to solve those IP models for big data observational studies will be computationally expensive.

### Contribution

In this work, we extend the robust causal inference testing method proposed by Morucci *et al.* [27] to handle large-scale observational studies with binary outcomes. To handle big data, first, we propose a robustness condition that identifies when a robust solution is possible and combines the maximization and minimization problems into a single problem. Second, we propose an efficient algorithm to calculate the test statistics for the robust condition. In addition, we propose two algorithms—one to solve the minimization problem and one to solve the maximization problem—for any condition that will show the degree of uncertainty for a selected number of matched pair. Finally, we implement the algorithms by testing the causal effect of HRRP to non-index readmissions using the State of California Patient Discharge Data and compare the computational efficiency with canonical IP solvers.

**Remark 1.** *Please note, by “Robust” we imply “Robust to the choice of matching method”: if* A *represents a set of all possible matching algorithms, a researcher choosing any algorithm* $A_{i}\in A$ *and testing a hypothesis of causal effect will get the same result if she has chosen algorithm* $A_{j\neq i}\in A$*. Also, we are considering matching as pre-processing and plan to achieve robust test result from a large-scale observational study for a given set of good matches* M *identified by any matching algorithm* $A_{i}\in A$*.*

### Causal inference with matching method and robust test

In the Rubin Causal Model, a sample unit *i* from a set of observations {1,2,⋯,n}∈S can have two outcomes or responses. The response $Y_{i}^{T}$ is called treatment response when the unit *i* receives certain treatment (*T* = 1) and control response when unit *i* does not receive treatment (*T* = 0). It is assumed that the treatment assignment of any unit does not interfere with the outcome of other units [36]. This assumption is commonly known as the Stable Unit Treatment Value Assumption (SUTVA). Under this assumption, the treatment effect on a sample unit i∈S is calculated as $TE_{i}=Y_{i}^{1}-Y_{i}^{0}$. However, it is impossible to observe the counterfactual scenario for the same sample [16]. Under a certain treatment regime T∈{0,1} and identical conditions, we can only observe $Y_{i}^{T=1}$ or $Y_{i}^{T=0}$ for sample *i*: $Y_{i}=T_{i}Y_{i}^{1}+(1-T_{i})Y_{i}^{0}$ [6,16]. Therefore, we cannot directly measure the treatment effect *TE* at an individual level. On the other hand, the causal inference literature offers a statistical solution to this fundamental problem by taking expectation over the observation set S, formally called *Average Treatment Effect (ATE)*.


$$\begin{array}{c} ATE=E\left[Y^{1}-Y^{0}|𝐗\right] \end{array}$$ (1)

The *ATE* as defined in Eq 1 provides the opportunity to divide S into the treatment group T when *T* = 1 and control group C when *T* = 0 such that (T∪C)=S and work with their expectations. So, we can construct the *ATE* as $E[Y^{1}|T=1]-E[Y^{0}|T=0]$ but, this form of *ATE* implicitly assumes that the potential responses are independent of treatment assignment: $Y_{i}^{1},Y_{i}^{0}⟂T,\forall i\in S$. Though this independence assumption holds in randomized experiments, in general, it does not hold for observational studies as the experimenter rarely has control over the treatment assignment process. This problem is solved by making an assumption known as Strong Ignorability [8]. Let 𝐗∈𝒳 and $𝐗\in {\mathbb{R}}^{k}$ be the set of pre-treatment background variables (covariates) which characterizes the observations. The strong ignorability assumption states that the potential responses are independent of treatment assignment when conditioned on the covariates: $Y_{i}^{1},Y_{i}^{0}⟂T|𝐗$ and every unit i∈S has a positive probability to receiving treatment: 0<Pr(T=1|𝐗=𝐱)<1. Another commonly used estimate of causal effect is *Average Treatment Effect on Treated (ATT)* which is defined under slightly relaxed assumption ($Y_{i}^{0}⟂T|𝐗$).


$$\begin{array}{c} ATT=E[(Y^{1}-Y^{0})|𝐗,T=1] \end{array}$$ (2)

Both of these estimates are prone to bias as the treatment assignment process is not random. In the matching method, an unbiased estimate of causal inference can be achieved if treatment unit t∈T is exactly matched with a control unit c∈C in terms of the covariate set 𝐗∈𝒳 [8]. However, in most of the applications, it is impossible to achieve exact matching [8,14,37,38]. A wide variety of matching methods are employed to make (*t*,*c*) pairs as similar as possible [8,14,39] or to find a subset of control group samples 𝒞⊆C that is similar to the treatment group samples 𝒯⊆T in the join*t* distribution of the covariate set **X** [30,37]. In this work, we consider one-to-one matching that aims to find a pair (t,c)⊆(T,C) that is matched (either exactly or by some user defined balance function) on a set of covariates 𝐗⊂𝒳.

Before explaining the difference between the classical method of causal inference [6,15,16] and the robust causal inference testing approach [27], let us define the set of good match M and the pair assignment variables *a*_*i*,*j*_.

**Definition 1.** (A set of Good Match) *A set of good match* M *includes treatment group samples* 𝒯⊆T *and control group samples* 𝒞⊆C *that satisfies certain covariate balance criteria defined under matching algorithm* $A_{i}\in A$*.*


$$\begin{array}{c} M:=\left\{(t,c)\in (𝒯\times 𝒞):t≃c|𝐗\right\} \end{array}$$

**Definition 2.** (Pair Assignment Operator) *The Pair Assignment Operator is a binary assignment variable* $a_{ij}\in \left\{0,1\right\}$ *where* $a_{ij}=1$ *if sample* $t_{i}\in 𝒯$ *is paired with a sample* $c_{j}\in 𝒞$ *and the pair* $(t_{i},c_{j})\in M$*;* $a_{ij}=0$ *otherwise.*

For a given set of possible matches M, we can perform hypothesis test in the following form with the null hypothesis being no causal effect and alternative being the opposite.


$$\begin{array}{c} {𝐇}_{0}^{ATE}:𝔼[Y^{1}-Y^{0}|𝐗]=0 \end{array}$$ (3)

$$\begin{array}{c} {𝐇}_{0}^{ATT}:𝔼[Y^{1}-Y^{0}|𝐗,T=1]=0 \end{array}$$ (4)

**Fig 2:**
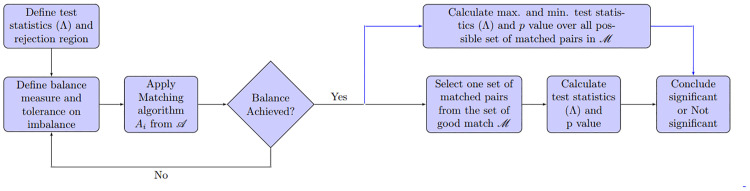
Comparison of matching for hypothesis testing under classical approach and robust approach [27]. Steps before covariate balance achievement remains same for each approach. In the remaining steps, Black arrows show the classical approach, and Blue arrows show the robust approach proposed in [27].

Under the classical approach of matching method, we can test these hypotheses first by defining a test statistic Λ, specifying an imbalance measure along with a tolerance limit on the imbalance. Then, we apply a matching algorithm $A_{i}\in A$ to find the set of good match M that satisfies the imbalance limit; otherwise we tune the allowable imbalance limit to generate M. Robust approach differs from the classical approach moving forward from here (see Fig 2). The classical approach picks one (out of many) possible combination of pairs from M and conducts the hypothesis test wherein, the robust approach calculate the maximum and minimum value of the test statistic $\left(\Lambda_{max},\Lambda_{min}\right)$ and corresponding p-values to explore all possible assignment combinations within M which does not increase imbalance under Definition 1. The test will be robust if both $\Lambda_{max}$ and $\Lambda_{min}$ produce same conclusion on the hypothesis. We formally define the Robust Test in Definition 3.

**Definition 3.** (Robust Test) *Let* α *be the level of significance set for the hypothesis* ${ℍ}_{0}$ *and* $\left(\Lambda_{max},\Lambda_{min}\right)$ *are the test statistics calculated from* M*, then, testing* **H**_0_ *is called* α*-robust if max*(p-value($\Lambda_{max}$), p-value($\Lambda_{min}$)) ≤ *α* *or min*(p-value($\Lambda_{max}$), p-value($\Lambda_{min}$)) > *α*. *Testing* **H**_0_ *is called absolute-robust when* p-value($\Lambda_{min}$) = p-value($\Lambda_{max}$)*.*

Calculating the test statistic Λ generates an integer programming model which is computationally expensive for large scale data (see Numerical Experiment section). In the following section, we propose a robustness condition following the Robust Test definition which will allow us to calculate a $\Lambda_{robust}=\Lambda_{min}=\Lambda_{max}$ for absolute-robust test and we can avoid solving two integer programming problems. From definition 3, it is clear that an absolute-robust test is always robust. In this work, we are interested in testing the hypothesis stated in Eqs (3),(4) for binary outcomes: Y∈{0,1} with the McNemar’s test [40] as proposed in [27].

### Robust McNemar’s test

McNemar’s test is the ideal candidate for testing hypothesis in Eqs (3,4) as it deals with one-to-one matched pairs. It operates on a 2 × 2 contingency table (see Table 1) and the test statistics under the null hypothesis assume that the marginal proportions are homogeneous. Among the four types of matched pairs, we are mainly interested in the discordant pairs $B=\sum_{i\in T}\sum_{j\in C}a_{ij}Y_{j}^{0}(1-Y_{i}^{1})$ and $C=\sum_{i\in T}\sum_{j\in C}a_{ij}Y_{i}^{1}(1-Y_{j}^{0})$ where *a*_*i*,*j*_ is the pair assignment operator defined in Definition 2. Here, *B* counts the number of pairs where treatment units has outcomes 0: *Y*^1^ = 0 and control units has outcomes 1: *Y*^0^ = 1 and *C* counts the discordant pairs where *Y*^1^ = 1 and *Y*^0^ = 0. Under the assumption of having at least 1 discordant pair: B+C≥1 we will use the test statistic Λ as defined in Eq (5) to test both hypotheses.


$$\begin{array}{c} \Lambda =\frac{B-C-1}{\sqrt{B+C}} \end{array}$$ (5)

**Table 1:** Contingency table of the outcomes of treatment and control observations.

	Treatment		
		Yes $\left(Y^{1}=1\right)$	No $\left(Y^{1}=0\right)$
	Yes $\left(Y^{0}=1\right)$	A	B
Control	No $\left(Y^{0}=0\right)$	C	D

Morucci *et al.* [27] proposed the following integer programming model that explores all possible assignment options and calculate maximum and minimum possible test statistics $\Lambda_{max}$ and $\Lambda_{min}$, respectively.


$$\begin{array}{c} {\text{Maximize/Minimize}}_{a}\;\Lambda (\text{a})=\frac{B-C-1}{\sqrt{B+C}} \end{array}$$

Subject to:


$$\begin{array}{c} \sum_{i\in T}\sum_{j\in C}a_{ij}Y_{j}^{0}(1-Y_{i}^{1})=B \end{array}$$ (6)

$$\begin{array}{c} \sum_{i\in T}\sum_{j\in C}a_{ij}Y_{i}^{1}(1-Y_{j}^{0})=C \end{array}$$ (7)

$$\begin{array}{c} B+C=m\;\text{(Total number of discordant pairs)} \end{array}$$ (8)

$$\begin{array}{c} \sum_{i\in T}a_{ij}\leq 1\;\forall j\;\text{(Choose at most one treatment observation)} \end{array}$$ (9)

$$\begin{array}{c} \sum_{j\in C}a_{ij}\leq 1\;\forall i\;\text{(Choose at most one control observation)} \end{array}$$ (10)

$$\begin{array}{c} \text{Additional user-defined covariate balance constraints to find }M \end{array}$$

$$\begin{array}{c} a_{ij}\in \left\{0,1\right\} \end{array}$$ (11)

The total number of discordant pair constraint in Eq (8) provides an opportunity to linearize the robust McNemar’s test model. We can calculate the maximum and minimum test statistic by solving the integer programming model iteratively for different values of *m* until either a robust solution is obtained under definition 3 or *m* cannot be increased further. For the latter case, we will not find a robust solution.

As it is shown in Table 1, *B* is the total number of untied responses when $Y_{i}^{1}=0$ is matched with $Y_{j}^{0}=1$. Similarly, *C* is total number of untied responses when $Y_{i}^{1}=1$ is matched with $Y_{j}^{0}=0$. Therefore, both $B,C\in {\mathbb{R}}^{+}$. Under this definition of *B* and *C*, we provide the following propositions on the objective function of the robust McNemar’s test and its optimal values.

**Proposition 1.** *The objective function* Λ(a)*, has the following properties:*

(i) *For any C > 0,* Λ(a) *is strictly increasing in B for* $B\in {\mathbb{R}}^{+}$
(ii) *For any* *B* ≥ 0, Λ(a) *is monotonically decreasing in C for* *C* ≥ 1 *and strictly decreasing for C > 1*

*Proof.* Let *C* > 0, then for any $B\in {\mathbb{R}}^{+}$, we have


$$\begin{array}{c} \frac{\partial \Lambda (\text{a})}{\partial B}=\frac{B+3C+1}{2(B+C{)}^{3/2}}>0 \end{array}$$ (12)

which implies Λ(a) is strictly increasing in *B* for a fixed *C*. Similarly, let B≥0, then for any *C* ≥ 1 we have,


$$\begin{array}{c} \frac{\partial \Lambda (\text{a})}{\partial C}=\frac{-3B-C+1}{2(B+C{)}^{3/2}}\leq 0 \end{array}$$ (13)

this proves the claims of Proposition 1. □

Before further discussion on Λ(a) and the optimality conditions, we introduce the following notations and definitions of maximum untied responses, for both *B* and *C*.

$\left|Y_{i}^{1}=1\right|$ is the number of treatment units in the matched set with positive outcome

$\left|Y_{i}^{1}=0\right|$ is the number of treatment units in the matched set with negative outcome

$\left|Y_{j}^{0}=1\right|$ is the number of control units in the matched set with positive outcome

$\left|Y_{j}^{0}=0\right|$ is the number of control units in the matched set with negative outcome

**Definition 4.** (Maximum type one discordant pair) *B*_*max*_ *is the maximum number of possible pairs between* $Y_{i}^{1}\in T$ *and* $Y_{j}^{0}\in C$ *where the treated observation has negative (“No”) outcome but the untreated (control) observation has positive (“Yes”) outcome, i.e.,*


$$\begin{array}{c} B_{max}=\min\left\{|Y_{j}^{0}=1|,|Y_{i}^{1}=0|\right\} \end{array}$$

**Definition 5.** (Maximum type two discordant pair) *C*_*max*_ *is the maximum number of possible pairs between* $Y_{i}^{1}\in T$ *and* $Y_{j}^{0}\in C$ *where the treated observation has positive (“Yes”) outcome but the untreated (control) observation has negative (“No”) outcome, i.e.,*


$$\begin{array}{c} C_{max}=\text{min}\left\{|Y_{i}^{1}=1|,|Y_{j}^{0}=0|\right\} \end{array}$$

For a fix value of *m*, the McNemar’s test model becomes linear and the objective functions become,


$$\begin{array}{c} \Lambda (\text{a})=\frac{1}{\sqrt{m}}(B-C-1) \end{array}$$ (14)

Using the property of Λ(a) explained in Proposition 1, we can find the optimal solution.

**Proposition 2.** *Let* *C* ≥ 1 *and denote m as the total number of discordant pairs, then the optimal pair* (C^*^, B^*^) *is given by:*


$$\begin{array}{c} \text{min:}\;(C^{*},B^{*})=\left\{ \begin{array}{cc} \left(C_{max},m-C_{max}\right) & \text{if}\;m>C_{max}\\ \left(m,0\right) & \text{if}\;m<C_{max} \end{array}\right.\\ \text{max:}\;(C^{*},B^{*})=\left\{ \begin{array}{cc} \left(m-B_{max},B_{max}\right) & \text{if}\;m>B_{max}\\ \left(0,m\right) & \text{if}\;m<B_{max} \end{array}\right. \end{array}$$

*Proof.* From Proposition 1, we know that Λ(a) is monotonically decreasing in C when *C* ≥ 1. Therefore, in the minimization problem, assignment will be made to maximize *C* until we are about to violate constraint *B* + *C* = *m*. When the total number of discordant pairs is set to *m* > *C*_*max*_, *C* will take the value of *C*_*max*_ and *B* will take the value of $m-C_{max}$ just to satisfy the total number of discordant pair constraints and the solution will be optimal. If *m* < *C*_*max*_, the new *C* = *m* and the minimum value will be achieved at *C* = *m* and *B* = 0.

Similarly, from Proposition 1, we know that Λ(a) is strictly increasing in *B* for any $B\in {\mathbb{R}}^{+}$. So, in the maximization problem, pair assignment will be made to maximize *B* within the feasible region. When the total number of discordant pair is set to *m* > *B*_*max*_, at optimal solution, *B* will take the value of *B*_*max*_ and *C* will take the value of $m-B_{max}$ just to stay in the feasible region. When *m* < *B*_*max*_, the *B* will take the value *m* and the maximum value will be achieved at *B* = *m* and *C* = 0. □

**Proposition 3.** *For the linear model, an absolute-robust estimate will be achieved if and only if the total number of discordant pair* $m=B_{max}+C_{max}$.

*Proof.* According to the proposed approach to the causal inference estimate, an absolute-robust estimate is achieved when $\Lambda (\text{a}{)}_{max}$ and $\Lambda (\text{a}{)}_{min}$ is equal. For the McNemar’s test model, the model becomes infeasible when *m* is set to $m>B_{max}+C_{max}$ as we can only have $B_{max}+C_{max}$ number of total untied responses. So feasible range of *m* is: $0<m\leq (B_{max}+C_{max})$.

To prove the Proposition 3, we first set *m* to it’s maximum value $B_{max}+C_{max}$. Using Proposition 2, in this case, the optimal solution for the $\Lambda (\text{a}{)}_{max}$ problem is: $B=B_{max}$, $C=m-B_{max}=C_{max}$ and the optimal solution for $\Lambda (\text{a}{)}_{min}$ problem is: $C=C_{max}$, $B=m-C_{max}=B_{max}$. So, for $m=B_{max}+C_{max}$ case, we get $\Lambda (\text{a}{)}_{max}=\Lambda (\text{a}{)}_{min}$ and the solution is absolute-robust.

Conversely, *m* can take any integer value in the range $0<m<(B_{max}+C_{max})$ which can lead to the following six cases. For each of the cases, we will find the optimal solution using Proposition 2.

- $0\leq B_{max}\leq C_{max}\leq m<(B_{max}+C_{max})$: The optimal solution for the minimization problem is $C=C_{max},B=m-C_{max}$ and the maximization problem is $C=m-B_{max},B=B_{max}$.
- $0\leq B_{max}<m\leq C_{max}<(B_{max}+C_{max})$: The optimal solution for the minimization problem is *C* = *m*, *B* = 0 and the maximization problem is $C=m-B_{max},B=B_{max}$.
- $0\leq C_{max}\leq B_{max}\leq m<(B_{max}+C_{max})$: The optimal solution for the minimization problem is $C=C_{max},B=m-C_{max}$ and the maximization problem is $C=m-B_{max},B=B_{max}$.
- $0\leq C_{max}\leq m\leq B_{max}<(B_{max}+C_{max})$: The optimal solution for the minimization problem is $C=C_{max},B=m-C_{max}$ and the maximization problem is *C* = 0, *B* = *m*.
- $0<m\leq C_{max}\leq B_{max}<(B_{max}+C_{max})$: The optimal solution for the minimization problem is *C* = *m*, *B* = 0 and the maximization problem is *C* = 0, *B* = *m*.
- $0<m\leq B_{max}\leq C_{max}<(B_{max}+C_{max})$: The optimal solution for the minimization problem is *C* = *m*, *B* = 0 and the maximization problem is *C* = 0, *B* = *m*.

For all of the above six cases, $\Lambda (\text{a}{)}_{max}\neq \Lambda (\text{a}{)}_{min}$, hence, the solution is not absolute-robust.

Therefore, the total number of discordant pairs *m* have to be $B_{max}+C_{max}$ to get an absolute-robust estimate. □

As we can see from the Proposition 2, the optimization problem has become a counting problem and can be solved efficiently for big data. However, the optimal solution calculated with Proposition 2 disregards the assignment constraints Eqs (9),(10) and additional user-defined constraints. To find the optimal solution using the result from Proposition 2 that is feasible, we take a two-step approach. At the first step, we handle the user-defined constraints to find a good set of match M as a pre-processing step. We can use any off-the-shelf matching algorithm for that purpose or define a separate pair assignment model with different covariate balance measure to find M. At the second step, we partition the set of good match M into 𝒫 partitions such that within a partition p∈{1,2,⋯,𝒫}, any treatment unit *t* can be matched with any control unit *c*. A formal definition of a partition is provided below.

**Definition 6.** (Partition of M) p⊂M *is a partition if any treatment unit* $t\in \left\{1,2,\cdots ,{𝒩}_{t}^{p}\right\}$ *is a good match to any control unit* $c\in \left\{1,2,\cdots ,{𝒩}_{c}^{p}\right\}$ *and* (t,c)∈M*. The reverse has to hold as well.*

Construction of partitions under Definition 6 ensures that only good matches are considered for assignment. In addition, Definition 4 calculates *B*_*max*_ by pairing negative outcomes of treatment units and positive outcomes of control units which inherently satisfies the pair assignment constraints Eqs (9,10). Similarly, we calculate *C*_*max*_ by assigning a pair between samples with positive treatment outcomes and negative control outcomes. Therefore, none of the treatment or control unit is used more than once in the pair assignment process which satisfies the pair assignment constraints Eqs (9,10).

Now, using the above mentioned results, we propose Algorithm 1 which identifies the robustness condition and corresponding absolute-robust test statistic $\Lambda (𝐚{)}_{robust}$.

**Algorithm 1: Absolute-robust test statistic $\Lambda (𝐚{)}_{robust}$ at robustness condition**

**Require:** Vector of outcomes $(Y^{1},Y^{0}{)}^{1},(Y^{1},Y^{0}{)}^{2},\cdots ,(Y^{1},Y^{0}{)}^{𝒫}$

$B_{max}\leftarrow 0$

$C_{max}\leftarrow 0$

**for** p=1:𝒫 **do**

$B^{p}\leftarrow min(|Y^{1}=0|,|Y^{0}=1|{)}^{p}$

$C^{p}\leftarrow min(|Y^{1}=1|,|Y^{0}=0|{)}^{p}$

$B_{max}\leftarrow B_{max}+B^{p}$

$C_{max}\leftarrow C_{max}+C^{p}$

**end for**

**return**

$\Lambda (𝐚{)}_{robust}=\frac{B_{max}-C_{max}-1}{\sqrt{B_{max}+C_{max}}}$

By the sketch of the Algorithm 1, it seems like we are only matching the discordant pairs and ignoring the other possible pair assignments in the data, which is not true. In Proposition 4, we show that we match maximum possible pairs.

**Proposition 4.** *Algorithm 1 ensures that the maximum possible pairs (t,c) are matched in* M*.*

*Proof.* To prove this Proposition, we only need to show that in any partition *p*, Algorithm 1 matches maximum possible pairs. Then, we can sum the maximum pair assignments across the partitions to achieve maximum possible pairs (*t*,*c*) assignment in M.

Lets consider a partition *p* where ${𝒩}_{t}^{p}$ denotes the number of treatment samples and ${𝒩}_{c}^{p}$ denotes the number of control samples. Hence, the maximum number of pairs we can assign in *p* is $min({𝒩}_{t}^{p},{𝒩}_{c}^{p})$. We will use ${𝒩}_{.}^{p+}$ to represent the number of samples with positive outcomes (*Y*^.^ = 1) and ${𝒩}_{.}^{p-}$ to represent the number of samples with negative outcomes (*Y*^.^ = 0). After assigning the discordant pairs ($B_{max}^{p}$) and ($C_{max}^{p}$) as we did in Algorithm 1, we are left with $\left({𝒩}_{t}^{p+}-C_{max}^{p})+({𝒩}_{t}^{p-}-B_{max}^{p}\right)$ treatment samples and $\left({𝒩}_{c}^{p+}-B_{max}^{p})+({𝒩}_{c}^{p-}-C_{max}^{p}\right)$ control samples. Now, we can assign the remaining treatment and control samples into the other two types of pairs *A* and *D* to their limit:


$$\begin{array}{c} A_{max}^{p}=min\left(({𝒩}_{t}^{p+}-C_{max}^{p}),({𝒩}_{c}^{p+}-B_{max}^{p})\right) \end{array}$$

$$\begin{array}{c} D_{max}^{p}=min\left(({𝒩}_{t}^{p-}-B_{max}^{p}),({𝒩}_{c}^{p-}-C_{max}^{p})\right) \end{array}$$

It is trivial to show that the for partition *p*,


$$\begin{array}{c} min({𝒩}_{t}^{p},{𝒩}_{c}^{p})=B_{max}^{p}+C_{max}^{p}+D_{max}^{p}+A_{max}^{p} \end{array}$$

**Fig 3:**
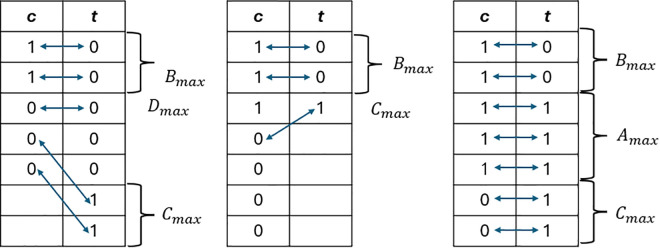
Example of maximum pair assignments between treatment and control group. *t* represents the treatment group and *c* represents the control group. An arrow connects a treatment unit with a control unit which forms a pair.

An example of maximum pair assignment is provided in Fig 3 where treatment outcomes (*t*) are sorted in ascending order and control outcomes (*c*) are sorted in descending order. In the left panel, we can have $min({𝒩}_{t}^{p},{𝒩}_{c}^{p})=5$ pairs at maximum. After assigning $B_{max}=2$ and $C_{max}=2$ according to Algorithm 1, we can assign only one pair to *D*_*max*_ and $A_{max}=0$. Therefore, we achieve the maximum number of pair assignments. We follow the similar procedure in the middle and right panels.

In the Algorithm 1, we calculate the absolute-robust test statistics at robustness condition which an experimenter can use to find the corresponding p-value and compare with a pre-defined level of significance α to make conclusion on the hypothesis of no causal relation. Decisions made in this process will be free of uncertainty and robust to the choice of matching algorithms. If different experimenters perform matching on same data using different matching algorithms but follow the above mentioned procedure, all of their conclusions will be exactly the same.

Regarding the computational complexity arises due to big data, our proposed algorithm only involves counting elements in vectors and few algebraic operations. The counting processing can be done with the summation of vectors as we are dealing with only binary outcomes: summation implies the total number of positive outcomes and we can calculate the negative outcomes by subtracting it from the size of the vector. In addition, we only need to solve the problem once—at robustness condition. Therefore, the proposed algorithm will be highly efficient for big data.

While the Algorithm 1 directly calculates test statistics at robustness condition, a researcher might be interested in exploring the degree of uncertainty in the causal inference test. She may want to see how the uncertainty changes towards the robust estimate with respect to the number of discordant pairs matched. For this purpose, we propose the following two algorithms (2, 3) following the result of Proposition 2.

**Algorithm 2: Maximizing the test statistics Λ(𝐚)**

**Require:** Vector of outcomes $(Y^{1},Y^{0}{)}^{1},\cdots ,(Y^{1},Y^{0}{)}^{𝒫}$ and increment in *m*: *I*

B,C←0

**while** $m\leq B_{max}+C_{max}$ **do**

**for** p=1:𝒫 **do**

**if** *m* < *B*_*max*_ **then**

$C^{p},B^{p}\leftarrow 0,m$

**else**

$C^{p},B^{p}\leftarrow m-B_{max},B_{max}$

**end if**

$B\leftarrow B+B^{p}$

$C\leftarrow C+C^{p}$

**if** (B+C)≥m **then**

break

**end if**

**end for**

m←m+I

**end while**

**return**

$\Lambda (𝐚{)}_{max}=\frac{B-C-1}{\sqrt{B+C}}$

**Algorithm 3: Minimizing the test statistics Λ(𝐚)**

**Require:** Vector of outcomes $(Y^{1},Y^{0}{)}^{1},\cdots ,(Y^{1},Y^{0}{)}^{𝒫}$ and increment in *m*: *I*

B,C←0

**while** $m\leq B_{max}+C_{max}$ **do**

**for** p=1:𝒫 **do**

**if** *m* < *B*_*max*_ **then**

$C^{p},B^{p}\leftarrow m,0$

**else**

$C^{p},B^{p}\leftarrow C_{max},m-C_{max}$

**end if**

$B\leftarrow B+B^{p}$

$C\leftarrow C+C^{p}$

**if** (B+C)≥m **then**

break

**end if**

**end for**

m←m+I

**end while**

**return**

$\Lambda (𝐚{)}_{min}=\frac{B-C-1}{\sqrt{B+C}}$

Now, to show the worst case time complexity for the proposed algorithms, we first define $q=\max_{p\in P}\left(|(Y^{1}{)}^{p}|,|(Y^{0}{)}^{p}|\right)$, where $(Y^{1}{)}^{p}$ and $(Y^{0}{)}^{p}$ denote the outcome vector for partition *p* for the treatment and control group, respectively. Then, by definition, the number of operations needed in Algorithm 1 for calculating the term $\min(|Y^{1}=0|,|Y^{0}=1|)$ is 2*q*. The time complexity becomes T(p)=p(4q+2). Therefore, Algorithm 1 has a time complexity of 𝒪(pq). Again, using the same definition of *q*, we can write $B_{\max}+C_{\max}\leq q+q=2q$. Since the time complexity for the loop (*p* = 1:*P*) is just 2 arithmetic operations, the overall time complexity of Algorithm 2 and 3 become $T(p)=\left(B_{\max}+C_{\max}\right)\left\{p(2)\right\}\leq (2q).(2p)=4pq$. Therefore, Algorithm 2 and 3 have a time complexity of 𝒪(pq).

### Numerical experiment

In this section, we present the efficiency of the proposed algorithms with data from the State of California Patient Discharge Database and address an interesting hypothesis on the effectiveness of the HRRP implemented in October 2012.

A hospital’s readmission rate is considered an important measure of its care quality. As noted, to increase the care quality and hold hospitals accountable, US Congress introduced the HRRP under the PPACA in 2012 [28]. The most important feature of this program is that the index hospital (the hospital that discharged the patient) is penalized if patients with pneumonia, congestive heart failure (CHF), and acute mayocardial infraction (AMI) are readmitted (to the index hospital or any other hospital) within 30 days of discharge. During the post HRRP period, the overall rate of readmission has been decreasing, which the proponents of HRRP are attributing to the success of the policy. However, in this period, readmission to different hospitals (non-index readmission) has been increasing [32,34]. Non-index readmissions have been found to be associated with longer lengths of stay and higher in-hospital mortality rates [35]. Hospitals are possibly discouraging patients seeking readmission to avoid penalties introduced by the HRRP. To examine the increase in non-index readmission post HRRP, we advance the following hypothesis and test it with the proposed algorithms with the level of significance α=0.05.

*H*_*0*_ *: HRRP has no causal relation with the increase in non-index readmission*

*H*_*1*_ *: HRRP has a positive causal relation with the increase in non-index readmission*

### Data description and covariate balance

In this research, we primarily used patient discharge data between 2010–2014 from California. We obtained this nonpublic data set from the California Office of Statewide Health Planning and Development (OSHPD), which collects in-patient data from California licensed hospitals. Each patient in this data set has a unique identifier that can be used to determine if a patient is readmitted. In addition, the data set also contains patient level information such as ICD-9 codes for clinical diagnosis, comorbidities, age, gender, discharge destination, patients’ Zip code, and insurance information. When a readmission was identified, we ascertained the destination hospital of that readmission. Then, a binary variable was created with 0 if the patient was readmitted to the same hospital or 1 if different hospital. To test the hypothesis, we used this variable as our outcome: *Y* = 1 if readmitted to a different hospital or *Y* = 0 if readmitted to the same hospital.

Moreover, the OSHPD data set was merged with publicly available data from the Centers for Medicare and Medicaid Services, American Association Annual Hospital Survey and the Area Resource file. From these additional data sources, we obtained important hospital-level information including teaching status (membership in the Council of Teaching Hospitals), ownership type (public, non-profit, investor owned), hospital size based on number of beds (small: below 100 beds, medium: 101–399 beds, and large: 400 and above beds) and hospital location (rural, metro). We also included a proxy for patient household incomes based on the median income of a patient’s residence Zip code. We divide the data into two sets: before and after October 1, 2012, the implementation date of HRRP. The treatment here is the implementation of HRRP, readmissions between February 1, 2010 and September 30, 2012 is considered as control group C (treatment *T* = 0) and readmissions from October 1, 2012 to November 30, 2014 is considered the treatment group T (treatment *T* = 1). To capture any potential readmission within 30 days of an index discharge, admissions before February 1, 2010 and beyond November 30, 2014 were excluded. A descriptive view of readmitted patients’ characteristics is presented in Table 2.

**Table 2:** Characteristics of readmitted patients in the State of California Patient Discharge Database from 2010 to 2014.

Variable	All Readmission	Index Hospital	Non-index Hospital	Before HRRP	After HRRP
**Readmitted Patients**	90553	67341	23212	53353	37200
**Demographic Characteristics**					
**Age**					
0-20	635 (0.70)	505 (0.75)	130 (0.56)	427 (0.8)	208 (0.56)
21-30	1073 (1.18)	717 (1.06)	356 (1.53)	566 (1.06)	507 (1.36)
31-40	2186 (2.41)	1471 (2.18)	715 (3.08)	1269 (2.38)	917 (2.47)
41-50	6336 (7.00)	4196 (6.23)	2140 (9.22)	3714 (6.96)	2622 (7.05)
51-65	21018 (23.21)	14470 (21.49)	6548 (28.21)	11950 (22.4)	9068 (24.38)
65 and above	59305 (65.49)	45982 (68.28)	13323 (57.4)	35427 (66.4)	23878 (64.19)
**Gender**					
Female	45124 (49.80)	34240 (50.80)	10884 (46.9)	27049 (59.94)	18075 (40.06)
Male	45429 (50.20)	33101 (49.20)	12328 (53.1)	26304 (57.9)	19125 (42.1)
**Household Income**					
Quartile 1	22428 (24.77)	15640 (23.23)	6788 (29.24)	13108 (24.57)	9320 (25.05)
Quartile 2	22629 (24.99)	16633 (24.70)	5996 (25.83)	13331 (24.99)	9298 (24.99)
Quartile 3	22450 (24.79)	17160 (25.48)	5290 (22.79)	13165 (24.68)	9285 (24.96)
Quartile 4	23046 (25.45)	17908 (26.59)	5138 (22.14)	13749 (25.77)	9297 (24.99)
**Clinical Characteristics**					
**Primary Diagnosis**					
CHF	50151 (55.40)	37404 (55.50)	12747 (54.9)	29351 (55.01)	20800 (55.91)
AMI	11917 (13.20)	8148 (12.10)	3769 (16.2)	6865 (12.87)	5052 (13.58)
Pneumonia	28485 (31.40)	21789 (32.40)	6696 (28.9)	17137 (32.12)	11348 (30.51)
**Charlson Comorbidity Index**					
Low (0–2)	35394 (39.09)	25884 (38.44)	9510 (40.97)	21282 (39.89)	14112 (37.94)
Medium (3–6)	51301 (56.65)	38454 (57.10)	12847 (55.35)	29850 (55.95)	21451 (57.66)
Medium High (7–10)	3396 (3.75	2628 (3.90)	768 (3.31)	1944 (3.64)	1452 (3.9)
High (10 and above)	462 (0.51)	375 (0.56)	87 (0.37)	277 (0.52)	185 (0.5)
**Hospital Characteristics**					
**Teaching Status**					
Teaching Hospital	10261 (11.30)	7706 (11.40)	2555 (11)	5882 (11.02)	4379 (11.77)
Non-teaching Hospital	80272 (88.70)	59635 (88.50)	20657 (89)	47471 (88.98)	32821 (88.23)
**Ownership Type**					
Non-profit Hospital	58592 (64.70)	45210 (67.10)	13382 (57.6)	34252 (64.2)	24340 (65.43)
Investor Hospital	17902 (19.80)	11389 (16.90)	6513 (28.1)	10839 (20.32)	7063 (18.99)
Public Hospital	14059 (15.50)	10742 (16.00)	3317 (14.3)	8262 (15.49)	5797 (15.58)
**Hospital Size**					
Small (below 100 beds)	4982 (5.50)	3453 (5.10)	1529 (6.6)	2979 (5.58)	2003 (5.38)
Medium (100–399 beds)	61167 (67.60)	45149 (67.10)	16018 (69)	36314 (68.06)	24853 (66.81)
Large (400 and above beds)	24404 (26.90)	18739 (27.80)	5665 (24.4)	14060 (26.35)	10344 (27.81)
**Hospital Location**					
Rural	2426 (2.68)	1809 (2.69)	617 (2.66)	1348 (2.53)	1078 (2.90)
Metro	88127 (97.32)	65532 (97.31)	22595 (97.34)	52005 (97.47)	36122 (97.10)

The entries in each cell is presented as Number of patients “N (%)” form. From February 1, 2010 to September 30, 2012 is considered “Before HRRP” period. From October 1, 2012 to December 31, 2014 is considered “After HRRP” period. CHF-Congestive Heart Failure, AMI-Acute Mayocardial Infraction.

We matched the patients based on the following covariates: age, gender, primary diagnosis, household income, Charlson Comorbidity Index, hospital location, hospital teaching status, hospital ownership status, and hospital size. We divided the covariates into two groups: 1) discrete and 2) continuous. The discrete covariates (i.e., gender, primary diagnosis, hospital location, hospitals’ teaching status, hospitals’ ownership status, and hospital size) are matched exactly. The continuous covariates (i.e., age, household income, and Charlson Comorbidity Index) were first divided into categories as shown in Table 2, then, the categories were matched exactly. This matching strategy resulted in 1822 partitions of data; within a partition any treatment sample can be matched with any control sample. The number of possible matched pairs in each partition can be calculated by taking the minimum number of treated or control samples in that partition. Among the 1822 partitions, we had 35,584 possible pairs. Though this matching approach seems ad hoc in nature, it is very similar to the well known method called Coarsed Exact matching (CEM) [30] with 1822 bins. Traditionally, CEM is implemented with a much lower number of bins due to the lack of common support between treatment and control groups but a higher number of bins makes for a finer covariate balance [41,42], which is the objective of any matching method. However, to implement the proposed algorithms, an experimenter is not limited to CEM or the matching method we used. Given a good set of matches created under Definition 1, we can always create the partitions under Definition 6.

### Experiment and result

To test the hypothesis *H*_*0*_, first, we performed the matching operations in R [43] to obtain matched sets. Then, using the matched sets of data, we calculated the test statistic $\Lambda (𝐚{)}_{max}$ and $\Lambda (𝐚{)}_{min}$ using the 1) optimization model with an Integer Programming solver and 2) with the proposed algorithms. The integer programming model was implemented in AMPL [44] and solved with the commercial solver CPLEX [45]. We implemented the Algorithm 1, 2, and 3 in R [43]. All the experiments were performed in a Dell Precision workstation with 64 GB RAM, Intel(R) Xeon(R) CPU E5-2670 v3 processor running at 2.30 GHz.

**Fig 4:**
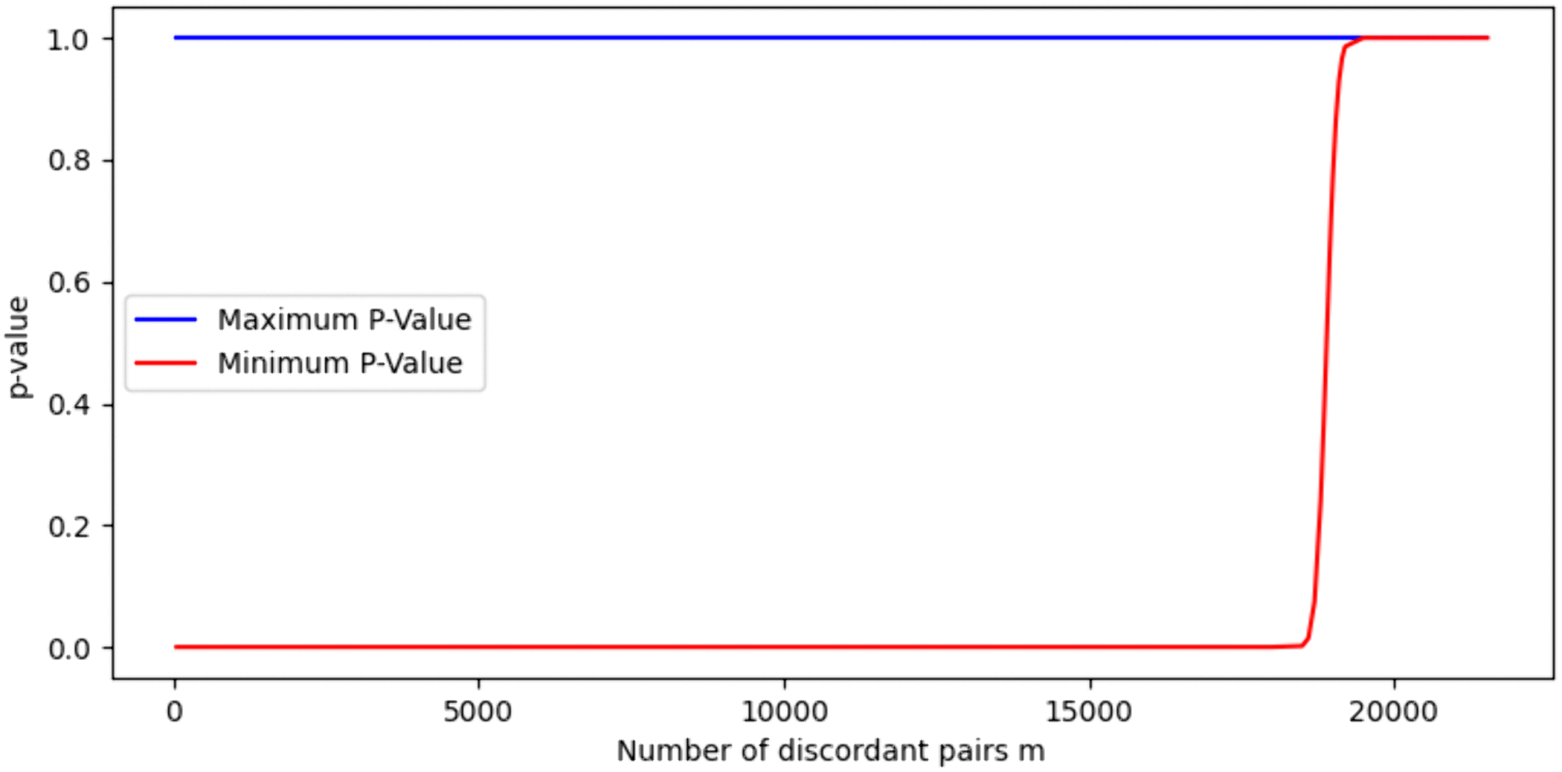
The range of p-value achievable for different number of discordant pairs *m.* The p-values were calculated using the test statistics presented in Table 3. The red line represents minimum possible p-value (corresponding to $\Lambda (𝐚{)}_{min}$) and the blue line represents the maximum possible p-value (corresponding to $\Lambda (𝐚{)}_{max}$).

Table 3 shows the comparison of solutions obtained using an optimization model with CPLEX iterating over different values of discordant pairs (*m*) and proposed algorithm at robustness condition. The range of p-value achievable corresponding to the test statistics $\Lambda (𝐚{)}_{max}$ and $\Lambda (𝐚{)}_{min}$ is presented in Fig 4. The proposed Algorithm 1 directly identifies the robustness condition which is $B_{max}=12082$ and $C_{max}=9448$ and calculates the absolute-robust test statistics $\Lambda (𝐚{)}_{robust}$. Including all four types of discordant pairs (*A*, *B*, *C*, *D*), Algorithm 1 generates 35,584 matched pairs. Regarding the efficiency of the hypothesis test, for a 5% level of significance we would need at least 942 pairs to have 90% power (calculated using result from Connor [46]) wherein we have 35,584 matched pairs. The computation time required by the proposed algorithm is very insignificant compared to the time required by the optimization model. A major implication of the robust McNemar’s test is that if the same experiment is conducted with as many as 15,000 discordant pairs, we can achieve any p-value between 0 to 1 (see Fig 4); some experimenter might reject the hypothesis and some might fail to reject the null hypothesis. Both experimenters, in this case, are right but their conclusions differed due to the fact that they choose different pairs. Any policy decision made using the matching method without considering this uncertainty has a possibility to fail.

**Table 3:** Test statistic Λ(𝐚) calculated using optimization model and algorithm 1.

	Optimization Model			Algorithm 1	
*m*	$\Lambda (𝐚{)}_{min}$	$\Lambda (𝐚{)}_{max}$	CPU time	Robustness Condition	CPU time
50	−7.21	6.93	918.69		
100	−10.10	9.90	982.23		
300	−17.38	17.26	1203.68		
500	−22.41	22.32	2037.37		
800	−28.32	28.25	2204.52		
1000	−31.65	61.60	2218.27		
5000	−70.72	70.69	2563.32		
10000	−88.97	99.99	2934.47		
15000	−31.82	74.82	2386.53		
20000	7.80	21.83	2659.94		
21000	14.51	21.83	2640.60		
21500	17.75	18.16	2219.23		
21530	17.94	17.94	3246.64	17.94^*^	1.13^*^

The optimization model is solved iteratively over different values of discordant pairs (*m*) until a robust solution is reached. ^*^Algorithm 1 identifies the robustness condition ($B_{max}=12082$ and $C_{max}=9448$) and calculates the test statistic for that condition only. CPU times are presented in seconds: time required to solve both minimization and maximization problem.

In regards to the hypothesis we made at the beginning of this section, we can reach a conclusion by using the p-value calculated at the robustness condition or the result from Table 3 and corresponding p-values from Fig 4. We can see that both the maximum and minimum p-value >α when we match more than 20,000 discordant pairs. Therefore, we fail to reject the null hypothesis of no causal effect and conclude that the HRRP is not a cause for increase in the non-index readmissions.

## Conclusion

Any policy decision or evaluation requires identifying the causal relation between policy alternatives and potential outcomes. Matching methods have become very popular in identifying such causal relations. However, in one-to-one matching, when we have multiple pair assignment options, matching method is vulnerable to uncertainty as the pair construction process does not consider outcomes. In this paper, we consider the integer programming model for robust causal inference testing approach with binary outcomes proposed by Morucci *et al.* [27] and develop scalable algorithms that can be used for large-scale observational studies. We identify a robustness condition that combines the maximization and minimization problem proposed in [27]. Instead of solving two computationally expensive integer programming models iteratively by increasing the number of discordant pairs until a robust estimate is achieved, we convert the problems into counting problems through a series of propositions. In addition, the proposed Algorithm 1 solves one problem instead of two separate problems and it is computationally efficient. Quadratic time complexity and the numerical experiment conducted on the State of California Patient Discharge Database show that the proposed algorithms are highly scalable. The numerical experiment demonstrates how the algorithm can be used to test for causal impacts from public policy initiatives – in this case a healthcare policy designed to reduce hospital readmissions – with observational data. We identify that the HRRP is not a cause of the increase of non-index readmissions, which has been shown to be associated with higher in-hospital mortality rate and a longer length of stay. Though the numerical experiment is performed with around 100,000 samples, the algorithms proposed in this paper can handle observational studies with millions of samples efficiently without further modification. In the future, we plan to develop similar robust causal inference testing algorithms with continuous outcomes for large-scale observational studies.

## Supporting information

S1 File Popularity of matching method. Google Scholar search results to show the popularity of matching method in causal inference.
(DOCX)
