# Supplementary material for: Robust policy evaluation from large-scale observational studies
Source: PLoS One. 2026 Jun 30;21(6):e0348228. doi: 10.1371/journal.pone.0348228 (PMC13318056; doi:10.1371/journal.pone.0348228)
Supplement: S1 File — Google Scholar search results to show the popularity of matching method in causal inference. (DOCX) [file pone.0348228.s001.docx]

**S1 File: Popularity of Matching**

A Google Scholar search for (matching OR match OR matched) AND (``causal inference" OR causality) resulted 486,000 articles. Accessed on March 01, 2019.
